# Supplementary material for: Polyketide synthase gene clusters in the ericoid mycorrhizal fungus Oidiodendron maius: genome-wide prediction, expression profiling, and functional characterization of knockout mutants
Source: Front Plant Sci. 2026 Sep 16;17:1924430. doi: 10.3389/fpls.2026.1924430 (PMC13623903; doi:10.3389/fpls.2026.1924430)
Supplement: Supplementary file 1 [file SupplementaryFile1.zip › Supplementary Materials/Supplementary Folder 1.zip!/Oidma1_AssemblyScaffolds_Repeatmasked/knownclusterblast/cluster51/jgi.p_Oidma1_35774_mibig_hits.html]

| MIBiG Protein | Description | MIBiG Cluster | MiBiG Product | % ID | % Coverage | BLAST Score | E-value |
| --- | --- | --- | --- | --- | --- | --- | --- |
| AAT65716.1 | putative\_monooxygenase | BGC0000629 | Aflatrem\_biosynthetic\_gene\_cluster | 37 | 98.0343980344 | 266 | 5.8e-71 |
| ACO31289.1 | PtmB3 | BGC0001140 | Platensimycin\_/\_platencin\_biosynthetic\_gene\_cluster | 30 | 99.5085995086 | 128 | 1.4e-29 |
| ALA99209.1 | CreL | BGC0001295 | Cremeomycin\_biosynthetic\_gene\_cluster | 32 | 81.8181818182 | 125 | 1.2e-28 |
| ADD82995.1 | PtnB3 | BGC0001156 | Platencin\_biosynthetic\_gene\_cluster | 29 | 99.5085995086 | 118 | 1.5e-26 |
| AAT65710.1 | putative\_monooxygenase | BGC0000629 | Aflatrem\_biosynthetic\_gene\_cluster | 27 | 102.948402948 | 106 | 1e-22 |
| CAP95403.1 |  | BGC0001404 | Sorbicillin\_biosynthetic\_gene\_cluster | 28 | 87.4692874693 | 91 | 2e-18 |
| ACZ57545.1 | predicted\_monooxygenase | BGC0000046 | Depudecin\_biosynthetic\_gene\_cluster | 23 | 96.8058968059 | 88 | 1.7e-17 |
| AGN71623.1 | salicylate\_hydroxylase | BGC0000027 | Azaphilone\_biosynthetic\_gene\_cluster | 26 | 86.9778869779 | 86 | 8.2e-17 |
| EED18000.1 | monooxygenase,\_putative | BGC0000154 | Stipitatic\_acid\_biosynthetic\_gene\_cluster | 26 | 86.7321867322 | 84 | 4.1e-16 |
| CAM34336.1 | putative\_FAD-depending\_monooxygenase | BGC0000242 | Lysolipin\_biosynthetic\_gene\_cluster | 26 | 92.628992629 | 77 | 3.8e-14 |
| CRG85571.1 | hypothetical\_protein | BGC0001402 | Cyclochlorotine\_biosynthetic\_gene\_cluster | 24 | 84.7665847666 | 73 | 5.5e-13 |
| AGO65986.1 | putative\_monooxygenase | BGC0000992 | Fusaridione\_A\_biosynthetic\_gene\_cluster | 31 | 42.7518427518 | 67 | 5.2e-11 |
| EHA28235.1 | hypothetical\_protein | BGC0001143 | Azanigerone\_biosynthetic\_gene\_cluster | 23 | 86.9778869779 | 66 | 1.2e-10 |
| CAP96443.1 | unnamed | BGC0000420 | Roquefortine\_C\_/\_meleagrin\_biosynthetic\_gene\_cluster | 26 | 42.0147420147 | 62 | 1.3e-09 |
| BAJ52699.1 | putative\_FAD-binding\_monooxygenase | BGC0000222 | FD-594\_biosynthetic\_gene\_cluster | 25 | 84.5208845209 | 61 | 2.8e-09 |
| ABB69742.1 | PlaT1 | BGC0000654 | Phenalinolactone\_biosynthetic\_gene\_cluster | 24 | 96.5601965602 | 51 | 2.3e-06 |
